# *Radiation Oncology* reviewer acknowledgement 2015

**DOI:** 10.1186/s13014-016-0610-1

**Published:** 2016-02-26

**Authors:** Claus Belka, Sarah M. Theissen

**Affiliations:** Department of Radiation Oncology, University Hospital of Munich, Marchioninistr. 15, 81377 Munich, Germany; BioMed Central, 236 Gray’s Inn Road, London, WC1X 8HB UK

## Abstract

The editorial team of *Radiation Oncology* would like to thank all our reviewers who have contributed to the journal in 2015.

Without the participation of skilful reviewers, no academic journal could succeed, and we are grateful to the committed individuals who have given their time and expertise to the peer review of manuscripts for *Radiation Oncology*. We look forward to your continued support in 2016.

**J Abayomi**

UK

**Takanori Abe**

Japan

**Aruna Abhyankar**

UK

**Yasser Abo-Madyan**

Germany

**Sonja Adebahr**

Germany

**Sebastian Adeberg**

Germany

**Vikram Adhikarla**

USA

**Sushma Agrawal**

India

**Markus Alber**

Denmark

**BM Aleman**

Netherlands

**Michelle Alonso-Basanta**

USA

**Shafak Aluwini**

Netherlands

**S Alvi**

UK

**Indu Ambudkar**

USA

**M Amsbaugh**

USA

**Nicolaus Andratschke**

Germany

**Stefano Arcangeli**

Italy

**Moran Artzi**

Israel

**Michael Atkinson**

Germany

**Marianne Aznar**

Denmark

**Houda Bahig**

Canada

**Kurt Baier**

Germany

**Elizabeth Baldini**

USA

**Hendrik Ballhausen**

Germany

**Mark Bangert**

Germany

**Carlos Barcenas**

USA

**Tomasz Barelkowski**

Germany

**M Baron Nelson**

USA

**Guido Baroni**

Italy

**Glenn Bauman**

Canada

**Wolfgang Baus**

Germany

**P Bayo**

Germany

**Jose Bazan**

USA

**Marcus Beck**

Germany

**Laurent Bedenne**

USA

**GL Beets**

Netherlands

**Jose Belderbos**

Netherlands

**ML Belli**

Italy

**J Berenson**

USA

**Jannette Beukema**

Netherlands

**Wolfgang Birkfellner**

Austria

**Marc Bischof**

Germany

**Pierre Blanchard**

France

**Oliver Blanck**

Germany

**Lennart Blomqvist**

Sweden

**Judit Boda-Heggemann**

Germany

**Raphael Bodensohn**

USA

**Beat Bojaxhiu**

Switzerland

**William Bonner**

USA

**JP Boström**

Germany

**Herbert Braselmann**

Germany

**S Breedveld**

Netherlands

**Michael Bremer**

Germany

**CL Brouwer**

Netherlands

**Michelle Brown**

Switzerland

**T Brown**

Australia

**Stephen Brown**

USA

**Thomas Brunner**

Germany

**J Buatti**

USA

**Grete Buchholz**

Germany

**Wilfried Budach**

Germany

**Michela Buglione**

Italy

**Irene A. Burger**

Switzerland

**Karl Butterworth**

UK

**Florian Büttner**

Germany

**Aras Emre Canda**

Turkey

**W Cao**

USA

**Joel Castelli**

France

**C Castoro**

Italy

**Jimmy Caudell**

USA

**Jean-Michel Caudrelier**

Canada

**Laura Cella**

Italy

**Giovanna Cenacchi**

Italy

**Laura Cervino**

USA

**S Chakraborty**

India

**Mark Ka Heng Chan**

Hong Kong

**Samuel Chao**

USA

**Naved Chaudhri**

Germany

**L Chien**

Taiwan

**Hyeon-Mih Cho**

South Korea

**Youngmin Choi**

South Korea

**Christos Chouaid**

France

**James Chow**

Canada

**F Cicone**

Italy

**Ilja Ciernik**

Germany

**Nikola Cihoric**

Switzerland

**Mario Ciocca**

Italy

**S Clemente**

Italy

**Brian Collins**

USA

**Sean Collins**

USA

**Anna Constantinescu**

Germany

**M Coskun-Breuneval**

Turkey

**Felipe Couñago**

Spain

**Luca Cozzi**

Italy

**Gilles Crehange**

France

**Richard Crevenna**

Austria

**Johannes Crezee**

Netherlands

**SB Crowe**

Australia

**Alessandra Curioni**

Switzerland

**Jakub Cvek**

Czech Republic

**Clemens Cyran**

Germany

**Brian Czito**

USA

**Giuseppe D’agostino**

Italy

**Alan Dal Pra**

Switzerland

**Sidsel Damkjaer**

Denmark

**Rolando M. D’Angelillo**

Italy

**Cyril Danjoux**

Canada

**S Dashti**

USA

**Niloyranjan Datta**

Switzerland

**P De Ieso**

Australia

**Reinhard Depping**

Germany

**Luc Dewit**

Netherlands

**K Dholam**

India

**Jennifer Dhont**

Belgium

**Alexander Dietrich**

Germany

**Sarah Differding**

Belgium

**Andreas Dinkel**

Germany

**Tcholpon Djuzenova**

Germany

**Sophie Dobiasch**

Germany

**Barbara Dobler**

Germany

**H Doi**

Japan

**Olivio Donati**

Switzerland

**A Donneys**

USA

**Wolfgang Dörr**

Germany

**Laura Doyle**

USA

**Guido Drexler**

Germany

**Laurence Dubrez**

France

**Yvonne Dzierma**

Germany

**F Ebner**

Germany

**Ardalan Ebrahimi**

Australia

**K Eda**

Turkey

**A Eisbruch**

USA

**Harriet Eldredge-Hindy**

USA

**John Eley**

USA

**Mostafa Elhaddad**

Egypt

**Olgun Elicin**

Switzerland

**Peter Ellis**

Canada

**Andrew Elson**

USA

**Wolfgang Enghardt**

Germany

**J Eriksen**

Denmark

**Iris Ernst**

Germany

**Birgit Ertl-Wagner**

Germany

**Marion Essers**

Netherlands

**Laura Evangelista**

Italy

**Khashayar Fakhrian**

Germany

**K Farr**

Denmark

**G Fastner**

Austria

**Dimitrios Fatouros**

Greece

**Giovanni Fattori**

Switzerland

**J Feddock**

USA

**Roman Fehr**

Germany

**Pascal Fenoglietto**

France

**MA Fernández-Seara**

Spain

**Petra Feyer**

Germany

**Jacob Finkelstein**

USA

**Alba Fiorentino**

Italy

**Michael Fix**

Switzerland

**Daniel Fleischmann**

Germany

**Ralf Floca**

Germany

**Tullio Florio**

Italy

**Robert Foerster**

Germany

**Antonella Fogliata**

Italy

**Emmanouil Fokas**

UK

**Davide Fontanarosa**

Netherlands

**M Foote**

Australia

**P Franco**

Italy

**Nicolaas Franken**

Netherlands

**Thorsten Frenzel**

Germany

**Benjamin Frey**

Germany

**Anna Friedl**

Germany

**Thomas Friedrich**

Germany

**Weihua Fu**

USA

**M Fujiwara**

Japan

**M Fukumoto**

Japan

**Simone Fulda**

Germany

**Guenther Gademann**

Germany

**Udo Gaipl**

Germany

**Razvan Galalae**

Germany

**Maria Antonietta Gambacorta**

Italy

**Gregory Gan**

USA

**Ute Ganswindt**

Germany

**Kevin Gardner**

USA

**Elisabetta Gargioni**

Germany

**Cristina Garibaldi**

Italy

**Tobias Gauer**

Germany

**Cengiz Gemici**

Turkey

**Michael Gensheimer**

USA

**Dietmar Georg**

Austria

**PC Gerszten**

USA

**Pirus Ghadjar**

Germany

**Sarbani Ghosh Laskar**

India

**David Gierga**

USA

**Suki Gill**

Australia

**Frank Giordano**

Germany

**Meredith Giuliani**

Canada

**Christoph Glanzmann**

Switzerland

**Daniel Gomez**

USA

**J Gómez-Millán**

Spain

**Holger Gottschlag**

Germany

**Gerhard Grabenbauer**

Germany

**Christian Graeff**

Germany

**Clemens Grassberger**

USA

**Peter Greer**

Australia

**Arne Gruen**

Germany

**M Gryzinski**

Poland

**G Guidi**

Italy

**JL Guinot**

Spain

**Marianne Guren**

Norway

**Cristina Gutierrez**

Spain

**Antonio Gutierrez**

Spain

**Christian Gutschow**

Switzerland

**N Gyldenkerne**

Denmark

**Rick Haas**

Netherlands

**Daniel Habermehl**

Germany

**Gregor Habl**

Germany

**Fred Hacker**

USA

**Marlen Haderlein**

Germany

**Bruce Haffy**

USA

**Matthias Häfner**

Germany

**Ester Hammond**

UK

**Jean-Michel Hannoun-Levi**

France

**Matthew Harkenrider**

USA

**Matthias Hartmann**

Switzerland

**Takayuki Hashimoto**

Japan

**Henrik Hauswald**

Germany

**Maria Hawkins**

UK

**S He**

China

**Rolf A. Heckemann**

UK

**Christine Heinrich**

Germany

**Miachael Henke**

Germany

**Willers Henning**

USA

**Klaus Herfarth**

Germany

**Julia Heß**

Germany

**Shuichi Hironaka**

Japan

**Ying Hitchcock**

USA

**Holger Hof**

Germany

**Jan Hofmaier**

USA

**W Hofstetter**

USA

**Yoshitaka Honma**

Japan

**S Hoogcarspel**

Netherlands

**Mohammad B Hossain**

USA

**Stephan Huber**

Germany

**Florence Huguet**

France

**David Hui**

USA

**Michael Ingrisch**

Germany

**Peter Inskip**

USA

**Edy Ippolito**

Italy

**Satoshi Ishikura**

Japan

**Hiromichi Ishiyama**

Japan

**Fumiaki Isohashi**

Japan

**Rolf D. Issels**

Germany

**Julian Jacob**

France

**Aaron Jacobs**

USA

**Stefan Janssen**

Switzerland

**Verena Jendrossek**

Germany

**Alexandra Jensen**

Switzerland

**Xun Jia**

USA

**Hosang Jin**

USA

**Keiichi Jingu**

Japan

**Lang Jingyi**

China

**Mirjana Josipovic**

Denmark

**An Jusheng**

China

**Johannes Kaanders**

Netherlands

**Ulf Kahlert**

Germany

**A Kakria**

India

**Sanjeeva Kalva**

USA

**MK Kam**

USA

**Florian Kamp**

Germany

**Severin Kampfer**

Germany

**Josephine Kang**

USA

**Steffi Kantz**

Germany

**Tom Karagiannis**

Australia

**I Karam**

Canada

**Sonja Katayama**

Germany

**David Kaul**

Germany

**Chul-Seung Kay**

South Korea

**T Kehwar**

USA

**Monika Keller**

Germany

**M Kent**

USA

**Lucyna Kepka**

Poland

**Oliver Kepp**

France

**Sarah Kerns**

USA

**Ki Chang Keum**

South Korea

**R Keus**

Netherlands

**E Khalil**

Egypt

**M Khan**

USA

**Nikolai Khodarev**

USA

**Divya Khosla**

India

**Philipp Kickingereder**

USA

**YH Kim**

North Korea

**NamKyu Kim**

South Korea

**Yong Bae Kim**

South Korea

**Grace Kim**

USA

**Tari King**

USA

**Rainer Klement**

Germany

**Stephan Kloeck**

Switzerland

**Sebastian Klüter**

Germany

**Shirley Knauer**

Germany

**Stefan Knippen**

Germany

**Thomas Koch**

Germany

**Martin Kocher**

Germany

**Fumitaka Koga**

Japan

**David Koh**

USA

**H Kok**

Netherlands

**Vassilis Kouloulias**

Greece

**Mechthild Krause**

Germany

**Robert Krempien**

Germany

**Marco Krengli**

Italy

**Jan Kriz**

Germany

**David Krug**

Germany

**Kevin Krull**

USA

**Marko Krznaric**

UK

**Sebastian Kuger**

Germany

**A Kunwar**

Canada

**Joseph Kurian**

USA

**George Kyrgias**

Greece

**Guillaume Landry**

Germany

**Stephanie Lang**

Switzerland

**J van Lanschot**

Netherlands

**Jung-Il Lee**

South Korea

**Ik Jae Lee**

South Korea

**Andrew Lee**

USA

**P Leone**

USA

**Vera Levina**

USA

**Antonin Levy**

France

**John Lewis**

USA

**Baosheng Li**

China

**Minglun Li**

Germany

**Jianbin Li**

China

**Karen Liby**

USA

**Do Hoon Lim**

South Korea

**M Lim**

South Korea

**S Lin**

USA

**Jin-Ching Lin**

Taiwan

**Katja Lindel**

Germany

**Claudia Linsenmeier**

Switzerland

**Jun Liu**

China

**Chao Liu**

USA

**Kristina Loessl**

Switzerland

**Frank Lohr**

Germany

**A Lollert**

Germany

**M Lopes**

Brazil

**Jose López Torrecilla**

Spain

**Alexander Louie**

Canada

**Dorota Lubgan**

USA

**Johannes Lutterbach**

Germany

**Stephen Lutz**

USA

**J Ma**

China

**Andreas Mack**

Switzerland

**K Maeda**

Japan

**Angelo Maggio**

Italy

**Umesh Mahanshetty**

India

**Cornelius Maihöfer**

Germany

**Philippe Maingon**

France

**Raymond Mak**

USA

**Venkata Manem**

Canada

**M Maraldo**

Denmark

**Boris Margulis**

Russia

**Maurie Markman**

USA

**Simone Marnitz**

Germany

**J Marsh**

USA

**David Mathieu**

Canada

**Christiane Matuschek**

Germany

**M McDonald**

USA

**Andrew McDonald**

USA

**Icro Meattini**

Italy

**Alejandra Mendez Romero**

Netherlands

**Valeria Mercadante**

UK

**P Metcalfe**

Australia

**Bernhard Meyer**

Germany

**J Michel**

France

**Oliver Micke**

Germany

**Ben Mijnheer**

Netherlands

**Michael Milano**

USA

**Giuseppe Minniti**

Italy

**Raymond Miralbell**

Switzerland

**Rene-Olivier Mirimanoff**

Switzerland

**Martin Misslbeck**

Germany

**Yasuyoshi Miyata**

Japan

**Anouchka Modesto**

France

**Raphaël Moeckli**

Switzerland

**Simone Moertl**

Germany

**Tan-Lucien Mohammed**

USA

**Majid Mohiuddin**

USA

**Konrad Mohnike**

Germany

**Michael Molls**

Germany

**F Moretto**

Italy

**Alessio Giuseppe Morganti**

USA

**Lutz Moser**

Germany

**Thorsten Moser**

Germany

**Filipe Moura**

Portugal

**Michael Moyers**

USA

**Petra Mozes**

Germany

**Nikola Mueller**

Germany

**Arndt-Christian Müller**

Germany

**Klaus Müller**

Germany

**Reinhold Müller**

Germany

**Olaf Nairz**

Austria

**N Nakajima**

Japan

**Pierina Navarria**

Italy

**Dunlap Neal**

USA

**VP Nelamangala Ramakrishnaiah**

India

**Nicole Nesvacil**

Austria

**Nam Nguyen**

USA

**R. Charles Nichols**

USA

**Geoffrey Nichols**

USA

**Giorgia Nicolini**

USA

**Carsten Nieder**

Norway

**Gabriele Niedermann**

Germany

**Peter Niehoff**

Germany

**Reinoud Nijhuis**

Germany

**A Nikoghosyan**

Germany

**K Nikolajek**

Germany

**Maximilian Niyazi**

Germany

**S Nougaret**

France

**Tuathan O’Shea**

UK

**Markus Oechsner**

Germany

**Christian Okoye**

USA

**Robert Olson**

Canada

**Ester Orlandi**

Italy

**Michael Orth**

Germany

**Feras Oskan**

Germany

**Piet Ost**

Belgium

**O Oyeyemi**

Nigeria

**Y Ozawa**

Japan

**Mahmut Ozsahin**

Switzerland

**Timothy Padera**

USA

**Jianji Pan**

China

**Herb Pang**

Hong Kong

**Mike Partridge**

UK

**David Pasquier**

France

**Margarethus Paulides**

Netherlands

**Arnold Paulino**

USA

**Frank Paulsen**

Germany

**Ruth Paulssen**

Norway

**A Paumier**

France

**D Peiffert**

USA

**Vincent Pennaneach**

France

**Evangelia Peponi**

Greece

**Francesco Perri**

Italy

**Peter Petersen**

Denmark

**Heike Peulen**

Netherlands

**Asja Pfaffenberger**

Germany

**C Philippson**

Belgium

**B Pieters**

Netherlands

**Michael Pinkawa**

Germany

**M Pinkham**

Australia

**Tomasz Piotrowski**

Poland

**Ludwig Plasswilm**

Switzerland

**George Plataniotis**

UK

**Volker Platz**

Germany

**Buelent Polat**

Germany

**Csaba Polgar**

Hungary

**A Pontoriero**

Italy

**Ramachandran Prabhakar**

India

**Matthias Prall**

Germany

**Heru Prasetio**

Germany

**Jochen Prehn**

Ireland

**Brendan Prendergast**

USA

**Vesna Prokic**

Germany

**Martin Pruschy**

Switzerland

**Pedro Puig**

Spain

**Paul Martin Putora**

Switzerland

**Florian Putz**

Germany

**Sharon Qi**

USA

**Cornelis Raaijmakers**

Netherlands

**Ulla Ramm**

Germany

**Andrew Rankin**

USA

**Coen Rasch**

Netherlands

**Paul Read**

USA

**Peter Reher**

Australia

**Michael Reiner**

Germany

**Marco Riboldi**

Italy

**Patrick Richard**

USA

**Andreas Richter**

Germany

**Daniel Richter**

Germany

**Juliane Rieber**

Germany

**Harald Rief**

Germany

**Stefan Rieken**

Germany

**Oliver Riesterer**

Switzerland

**Andreas Rimner**

USA

**Mark Rivard**

USA

**Mack Roach**

USA

**David Roberge**

Canada

**Stephen Roberts**

UK

**Nathalie Rochet**

Germany

**Franz Rödel**

Germany

**Dirk Roggenbuck**

Germany

**Jesse Roman**

USA

**Nicola Rosenfelder**

UK

**Stephan Roth**

Germany

**Kai Rothkamm**

Germany

**Carmen Rubio**

Spain

**Justine Rudner**

Germany

**Tatjana Rundek**

USA

**Elvio Grazioso Russi**

Italy

**Sebastia Sabater**

Spain

**Mohammadsaeed Saboori**

Germany

**Arjun Sahgal**

Canada

**Joseph Salama**

USA

**Ladan Saleh-Ebrahimi**

Germany

**Henning Salz**

Germany

**D Sapkaroski**

Australia

**Ryohei Sasaki**

Japan

**Dörthe Schaue**

USA

**Heike Scheithauer**

Germany

**Devin Schellenberg**

Canada

**Michel Schlienger**

France

**Thomas Schmid**

Germany

**Heinz Schmidberger**

Germany

**M Schmidt**

USA

**Uwe Schneider**

Switzerland

**Michael Scholz**

Germany

**Ed Schwalbe**

UK

**V Scotti**

Italy

**Felix Sedlmayer**

Austria

**Petra Seibold**

Germany

**Max Seidensticker**

Germany

**A Sekine**

Japan

**Robert Semrau**

Germany

**Sabine Semrau**

Germany

**Suresh Senan**

Netherlands

**Matteo Seregni**

Italy

**A Serna**

Spain

**Marco Serpa**

Germany

**D Severin**

Canada

**Daya Nand Sharma**

India

**Gregory Sharp**

USA

**J Sheehan**

USA

**David Shepard**

USA

**Helen Shih**

USA

**T Showalter**

USA

**Pei-Wei Shueng**

Taiwan

**David Shultz**

USA

**Frank-Andre Siebert**

Germany

**Anurag Singh**

USA

**Ira-Ida Skvortsova**

Austria

**Nicholas Slevin**

UK

**Robert Smee**

Australia

**Wendy Smith**

Canada

**Matthias Soehn**

Germany

**Dennis Sohn**

Germany

**Y Song**

China

**Jan-Jakob Sonke**

Netherlands

**Yu Yang Soon**

Singapore

**Stefan Speer**

Germany

**Michael Spiotto**

USA

**P Srivastava**

India

**Kevin Stephans**

USA

**Florian Sterzing**

Germany

**Florian Stieler**

Germany

**Alison Stillie**

UK

**Sandro Stoeckli**

Switzerland

**Guy Storme**

Belgium

**Christoph Straube**

Germany

**Tino Streller**

Switzerland

**Vratislav Strnad**

Germany

**Carmen Stromberger**

Germany

**Gabriela Studer**

Switzerland

**Martin Stuschke**

Germany

**Kristin Stützer**

Germany

**Isolde Summerer**

Germany

**K Sun**

China

**K Suzuki**

Japan

**O Suzuki**

Japan

**SG Swarts**

USA

**Marta Switlyk**

Norway

**Ghazaleh Tabatabai**

Germany

**P Tai**

Canada

**Oike Takahiro**

Japan

**M Takeuchi**

Japan

**Yamei Tang**

China

**Mohmad Ashraf Teli**

India

**Loukas Thanos**

Greece

**Gudrun Theile**

Switzerland

**Christian Thieke**

Germany

**Hubert Thierens**

Belgium

**Christopher Thomas**

UK

**J Thompson**

Australia

**Zhen Tian**

USA

**Carmen Timke**

Germany

**Ingeborg Tinhofer**

Germany

**Manuel Todorovic**

Germany

**J Torok**

USA

**Almut Troeller**

Germany

**Klaus Trott**

Germany

**MH Truong**

USA

**Nikolaos Tselis**

Germany

**Alexandros Tsikkinis**

Switzerland

**Mitsutoshi Tsukimoto**

Japan

**Deniz Tural**

Turkey

**L Turcotte**

USA

**B Turkbey**

USA

**Mario Turri-Zanoni**

Italy

**Kristian Unger**

Germany

**Laura Van den Bergh**

Belgium

**Jacoba van der Zee**

Netherlands

**S van Dyk**

Australia

**L van Roozendaal**

Netherlands

**Jef Vandemeulebroucke**

Belgium

**N vanderWalde**

USA

**V Vassiliou**

Greece

**Peter Vaupel**

Germany

**Jirina Vavrova**

Czech Republic

**Wilko Verbakel**

Netherlands

**Frederik Verburg**

Germany

**Frank Verhaegen**

Netherlands

**M Vickers**

Canada

**Salvador Villa**

Spain

**N Vivekanandan**

India

**Erina Vlashi**

USA

**G Vogin**

France

**Dirk Vordermark**

Germany

**Masaru Wakatsuki**

Japan

**M Wakatsuki**

Japan

**A Walenkamp**

Netherlands

**Hui Wang**

China

**K Wang**

USA

**Luhua Wang**

China

**W Weichert**

Germany

**Robert Weinfurtner**

USA

**Michael Weller**

USA

**Stefan Welz**

Germany

**Thomas G. Wendt**

Germany

**R Werner**

Germany

**Moody Wharam**

USA

**Joachim Widder**

Netherlands

**Benedikt Wiestler**

Germany

**Tilo Wiezorek**

Germany

**Eric Winquist**

Canada

**Chris Winter**

Switzerland

**Lukas Winter**

Germany

**Florian Wirsdörfer**

Germany

**Holger Wirtz**

Germany

**Stefan Wirtz**

Germany

**Marnix Witte**

Netherlands

**Matthew Witten**

USA

**Barbara Witulla**

Germany

**Suzanne Wolden**

USA

**Ulrich Wolf**

Germany

**Jens Wölfelschneider**

Germany

**Rebecca Wong**

Canada

**Abraham Wu**

USA

**Q. Jackie Wu**

USA

**Hong-Gyun Wu**

North Korea

**A Wushou**

China

**Peter Wust**

Germany

**Ting Xu**

USA

**S Yahya**

UK

**Hideomi Yamashita**

Japan

**Jinzhong Yang**

USA

**Akihiro Yano**

Japan

**John Yarnold**

UK

**Yong Yin**

China

**Indra Yohannes**

Germany

**Sua Yoo**

USA

**K Younge**

USA

**Angelika Zabel-Du Bois**

Germany

**Bjorn Zackrisson**

Sweden

**Nikolaos Zamboglou**

Germany

**Youssef Zeidan**

USA

**M Zemanova**

Czech Republic

**Zhen Zhang**

China

**Rui Zhang**

USA

**Chong Zhao**

China

**Heming Zhen**

USA

**Thomas Zilli**

Switzerland

**Horst Zitzelsberger**

Germany

**Zachary Zumsteg**

USA

**Brigitte Zurl**

Austria

**Daniel Zwahlen**

Switzerland

**Felix Zwicker**

Germany

